# Crystal structures of two nickel compounds comprising neutral Ni^II^ hydrazone complexes and di­carb­oxy­lic acids

**DOI:** 10.1107/S2056989016020326

**Published:** 2017-01-06

**Authors:** Takumi Nakanishi, Osamu Sato

**Affiliations:** a Institute for Materials Chemistry and Engineering, Kyushu University, 744 Motoka, Nishi-ku, Fukuoka 819-0395, Japan

**Keywords:** crystal structure, hydrazone complex, hydrogen bond, di­carb­oxy­lic acid, halogen–halogen inter­actions, co-crystal

## Abstract

Two isostructural Ni^II^ compounds, bis­{*N*-[1-(pyridin-2-yl-κ*N*)eth­ylidene]pyridine-4-carbohydrazonato-κ^2^ *N*′,*O*}nickel(II)–2,5-di­chloro­terephthalic acid (1/1), [Ni(C_13_H_11_N_4_O)_2_](C_8_H_4_Cl_2_O_4_), and bis­{*N*-[1-(pyridin-2-yl-κ*N*)eth­ylidene]pyridine-4-carbohydrazonato-κ^2^ *N*′,*O*}nickel(II)–2,5-di­bromo­terephthalic acid (1/1), [Ni(C_13_H_11_N_4_O)_2_](C_8_H_4_Br_2_O_4_), were synthesized and their crystal structures determined. The pair of *N*,*N*′,*O*-tridentate *N*-[1-(pyridin-2-yl-κ*N*)eth­yl]pyridine-4-carbohydrazonate *L* ligands result in a *cis*-NiO_2_N_4_ octa­hedral coordination sphere for the metal ions. The asymmetric units consist of two half-mol­ecules of the di­carb­oxy­lic acids, which are completed by crystallographic inversion symmetry. In the respective crystals, the 2,5-di­chloro­terephthalic acid (H_2_Cl_2_TPA, **1-Cl**) mol­ecules form zigzag hydrogen-bonded chains with the [Ni(*L*)_2_] mol­ecules, with the hydrogen-bond distances in **1-Br** slightly longer than those in **1-Cl**. The packing is consolidated by aromatic π–π stacking between the di­carb­oxy­lic acid mol­ecules and terminal pyridine rings in [Ni(*L*)_2_] and short halogen–halogen inter­actions are also observed. The qualitative prediction of the H-atom position from the C—N—C angles of the terminal pyridine rings in *L* and the C—O distances in the carboxyl groups show that **1-Cl** and **1-Br** are co-crystals rather than salts.

## Abstract 2

Two Ni^II^ compounds forming zigzag chain structures through O—H⋯N hydrogen bonds between di­carb­oxy­lic acids and Ni^II^ complexes were synthesized, and their structures were determined. They are co-crystals rather than salts.

## Chemical context   

Metal complexes based on 2-acetyl­pyridine isonicotinoyl­hydrazone (H*L*) have attracted considerable attention for the construction of supra­molecular materials (Servati Gargari *et al.*, 2015 ▸; Valipour *et al.*, 2016 ▸) and as functional complexes for applications in various biochemical fields (Ababei *et al.*, 2012 ▸; Chang, Jia, Xu, Xu *et al.*, 2015 ▸; Chang, Jia, Xu, Wu *et al.*, 2015 ▸). Moreover, the precursors of H*L*s and related hydrazone ligands have been used in the design of complexes stabilized by strong hydrogen bonds (Lemmerer *et al.*, 2010 ▸; Grobelny *et al.*, 2011 ▸; Aakeröy *et al.*, 2012 ▸; Cherukuvada & Nangia, 2012 ▸; Aitipamula *et al.*, 2009 ▸) and spin-crossover complexes (Hill *et al.*, 2010 ▸; Zhang *et al.*, 2010 ▸). Thus, it is possible that metal complexes with H*L* ligands could be applied in the design of various functional materials. We have reported spin-crossover compounds consisting of [Fe(*L*)_2_] and H_2_Cl_2_TPA, and of [Fe(*L*)_2_] and H_2_Br_2_TPA (H_2_Cl_2_TPA = 2,5-dichloroterephtalic acid, H_2_Br_2_TPA = 2,5-di­bromo­terephthalic acid), and it was observed that a one-dimensional zigzag hydrogen-bonding network involving short hydrogen bonds was formed between the [Fe(*L*)_2_] mol­ecules and di­carb­oxy­lic acids (Nakanishi & Sato, 2016 ▸). In this study, we present the crystal structures of the analogous Ni^II^ complexes, [Ni(*L*)_2_](H_2_Cl_2_TPA) (**1-Cl**) and [Ni(*L*)_2_)](H_2_Br_2_TPA) (**1-Br**).
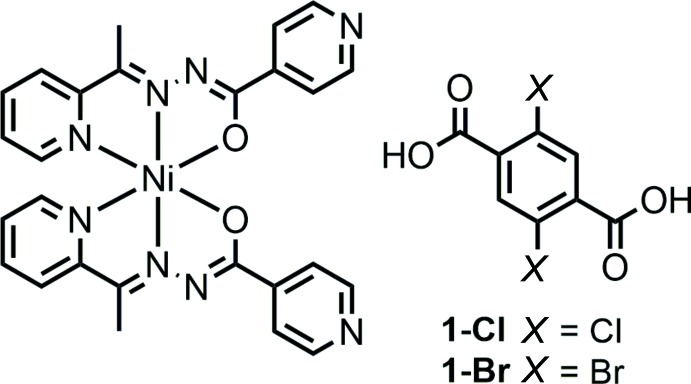


## Structural commentary   

**Figure 1:**
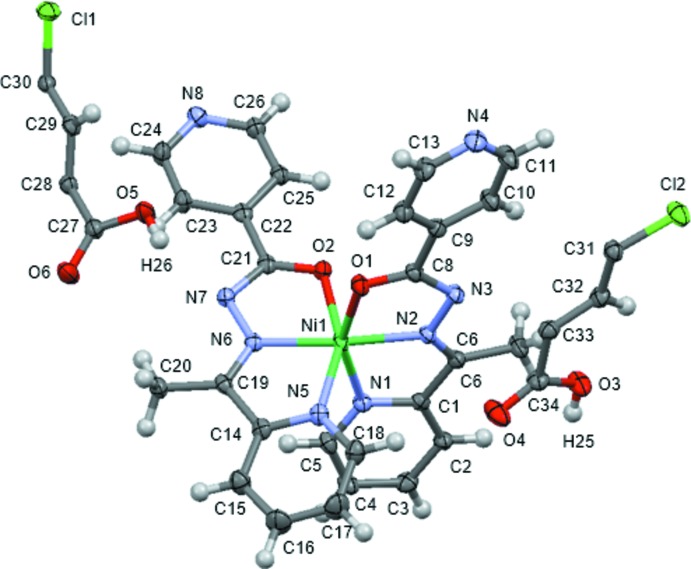
The asymmetric unit of [Ni(*L*)_2_](H_2_Cl_2_TPA) (**1-Cl**), shown with 50% probability displacement ellipsoids.

The mol­ecular structures of **1-Cl** and **1-Br** are displayed in Figs. 1 ▸ and 2 ▸, respectively. The crystal structures of **1-Cl** and **1-Br** are isostructural with each other: the asymmetric unit comprises one [Ni(*L*)_2_] mol­ecule and two half di­carb­oxy­lic acid mol­ecules, which are completed by crystallographic inversion symmetry. These are hereafter designated as (H_2_ *X*_2_TPA)_*A*_ with O3 and O4 and (H_2_ *X*_2_TPA)_*B*_ with O5 and O6 (*X* = Br, Cl). The pair of *N*,*N*′,*O*-tridentate *L* ligands generate a *cis*-NiO_2_N_4_ octa­hedron in each case.

Unfortunately, the hydrogen-atom positions in the hydrogen-bonding network could not be determined from difference Fourier maps. However, the positions of hydrogen atoms involved in hydrogen bonds could be predicted qualitatively from the C—N—C angles of the terminal pyridine ring in *L* and the C—O bond lengths of the carboxyl group in H_2_ *X*_2_TPA. The coordination distances and angles related to the hydrogen-bonding network are listed in Table 1 ▸. The C13–N4–C11 and C24–N8–C26 bond angles in **1-Cl** are 117.4 (2)° and 118.8 (2)°, respectively, and these are categorized as being part of a non-protonated pyridine ring (Bis & Zaworotko, 2005 ▸). Moreover, the C34—O3 and C27—O5 distances in **1-Cl** are 1.316 (3) and 1.306 (3) Å, respectively, and these clearly correspond to a protonated carb­oxy­lic acid (Bis & Zaworotko, 2005 ▸). These results indicate that the hydrogen atoms involved in hydrogen bonds are mainly located on the H_2_ *X*_2_TPA side. Therefore, it could be concluded that **1-Cl** is a co-crystal, comprising neutral [Ni(*L*)_2_] complexes and H_2_Cl_2_TPA mol­ecules, rather than a salt. The same conclusion can be drawn concerning **1-Br** (Table 2 ▸).

## Supra­molecular features   

**Figure 3:**
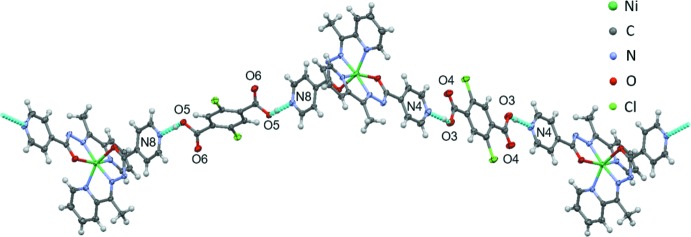
The mol­ecular arrangement of [Ni(*L*)_2_] and H_2_Cl_2_TPA in a zigzag hydrogen-bonded chain.

The mol­ecular arrangement in the hydrogen-bonding network in **1-Cl** is shown in Fig. 3 ▸. It was confirmed that [Ni(*L*)_2_] forms a one-dimensional zigzag hydrogen-bonding network with H_2_ *X*_2_TPA *via* the terminal py ring in *L*. In addition, in each case the two H_2_ *X*_2_TPA mol­ecules can be differentiated from one another in terms of the hydrogen-bond distance in the hydrogen-bonding chain; the (H_2_ *X*_2_TPA)_*A*_ mol­ecule forms a long hydrogen bond [N4⋯O3 = 2.679 (3) Å] and the (H_2_ *X*_2_TPA)_*B*_ mol­ecule forms a shorter hydrogen bond [N8⋯O5 = 2.547 (3) Å] (Table 3 ▸). The carboxyl groups in each H_2_ *X*_2_TPA mol­ecule are related by inversion, hence exhibiting the same hydrogen bonds at each end of the mol­ecule (Fig. 3 ▸). The hydrogen-bond distance N8⋯O5 [2.547 (3) Å] is relatively short, but not comparable with the distances observed in the organic compounds that exhibit proton migration (Steiner *et al.*, 2001 ▸; Cowan *et al.*, 2003 ▸, 2005 ▸). The hydrogen bond distances N4⋯O3 and N8⋯O5 in **1-Br** are 2.706 (4) and 2.557 (4) Å (Table 4 ▸), respectively, and these are clearly longer than the equivalent bonds in **1-Cl**; the same tendency was confirmed when comparing [Fe(*L*)_2_](H_2_Cl_2_TPA) and [Fe(*L*)_2_](H_2_Br_2_TPA) (Nakanishi & Sato, 2016 ▸).

**Figure 4:**
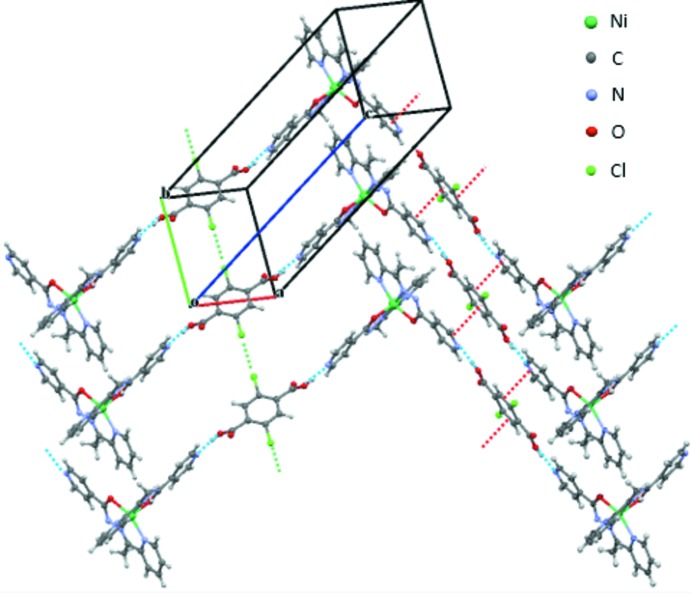
An overview of the the two-dimensional supra­molecular network comprising hydrogen bonds, π–π inter­actions and halogen–halogen inter­actions.

Another inter­molecular inter­action in each H_2_ *X*_2_TPA, a π–π inter­action, is found between the (H_2_ *X*_2_TPA)_*A*_ mol­ecule and the terminal pyridine ring in [Ni(*L*)_2_]. Furthermore, the (H_2_ *X*_2_TPA)_*B*_ mol­ecules form halogen–halogen inter­actions with adjacent (H_2_ *X*_2_TPA)_*B*_ mol­ecules [**1-Cl**: Cl1⋯Cl1^i^ = 3.435 (1) Å, C30–Cl1⋯Cl1^i^ = 129.64 (9)°; **1-Br**: Br1⋯Br1 = 3.5240 (8) Å, C30—Br1⋯Br1^i^ = 125.3 (1)°; symmetry code: (i) 1 – *x*, −1 – *y*, –*z*] as observed in an overview of the crystal structure (Fig. 4 ▸).

## Synthesis and crystallization   

[Ni(*L*)_2_](H_2_Cl_2_TPA) (**1-Cl**)

H*L* was synthesized according to the published procedure (Ababei *et al.*, 2011 ▸). H*L* (48 mg, 0.20 mmol) was dissolved in methanol (40 ml); then, NiCl_2_·6H_2_O (24 mg, 0.10 mmol) was added to the solution. Following this, H_2_Cl_2_TPA (24 mg, 0.10 mmol) was added to the solution. The mixture was stirred for 30 s. Subsequently, the solution was evaporated in air over a period of several days. Plate-shaped brown crystals were obtained.

[Ni(*L*)_2_](H_2_Br_2_TPA) (**1-Br**)

The synthesis procedure for **1-Br** is similar to that for **1-Cl**, except for the use of H_2_Br_2_TPA instead of H_2_Cl_2_TPA. Plate-shaped brown crystals were obtained.

## Refinement   

Crystal data, data collection and structure refinement details are summarized in Table 5 ▸. The hydrogen atoms connected to the carbon atom were treated using a riding model: C—H (aromatic) = 0.96 Å and C—H (meth­yl) = 0.98 Å. The hydrogen atoms involved in hydrogen bonds were also treated as riding with O—H = 0.84 Å.

## Supplementary Material

Crystal structure: contains datablock(s) global, 1-Cl, 1-Br. DOI: 10.1107/S2056989016020326/hb7631sup1.cif

Structure factors: contains datablock(s) 1-Cl. DOI: 10.1107/S2056989016020326/hb76311-Clsup2.hkl

Structure factors: contains datablock(s) 1-Br. DOI: 10.1107/S2056989016020326/hb76311-Brsup3.hkl

CCDC references: 1524024, 1524023

Additional supporting information:  crystallographic information; 3D view; checkCIF report

## Figures and Tables

**Figure 2:**
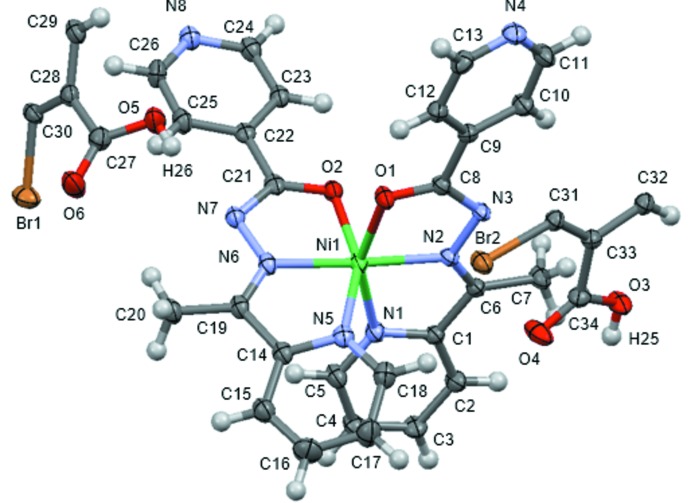
The asymmetric unit of [Ni(*L*)_2_](H_2_Br_2_TPA) (**1-Br**), shown with 50% probability displacement ellipsoids.

**Table 1:** Selected geometric parameters (Å, °) for **1-Cl** [Chem scheme1]

Ni1—O1	2.0870 (17)	Ni1—N6	1.985 (2)
Ni1—O2	2.1099 (19)	C34—O3	1.316 (3)
Ni1—N1	2.106 (2)	C34—O4	1.206 (3)
Ni1—N2	1.990 (2)	C27—O5	1.306 (3)
Ni1—N5	2.110 (2)	C27—O6	1.216 (3)
			
C11—N4—C13	117.4 (2)	C24—N8—C26	118.8 (2)

**Table 2:** Selected geometric parameters (Å, °) for **1-Br** [Chem scheme1]

Ni1—O1	2.079 (2)	Ni1—N6	1.983 (3)
Ni1—O2	2.118 (3)	C34—O3	1.324 (4)
Ni1—N1	2.110 (3)	C34—O4	1.207 (4)
Ni1—N2	1.986 (3)	C27—O5	1.300 (4)
Ni1—N5	2.116 (3)	C27—O6	1.223 (4)
			
C11—N4—C13	117.1 (3)	C24—N8—C26	118.2 (3)

**Table 3:** Hydrogen-bond geometry (Å, °) for **1-Cl** [Chem scheme1]

*D*—H⋯*A*	*D*—H	H⋯*A*	*D*⋯*A*	*D*—H⋯*A*
O3—H25⋯N4^i^	0.84	1.84	2.679 (3)	177
O5—H26⋯N8^ii^	0.84	1.71	2.547 (3)	176

Symmetry codes: (i) 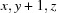; (ii) 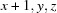.

**Table 4:** Hydrogen-bond geometry (Å, °) for **1-Br** [Chem scheme1]

*D*—H⋯*A*	*D*—H	H⋯*A*	*D*⋯*A*	*D*—H⋯*A*
O3—H25⋯N4^i^	0.84	1.87	2.706 (4)	178
O5—H26⋯N8^ii^	0.84	1.72	2.557 (4)	172

Symmetry codes: (i) 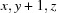; (ii) 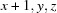.

**Table 5:** Experimental details

	**1-Cl**	**1-Br**
Crystal data		
Chemical formula	[Ni(C_13_H_11_N_4_O)_2_](C_8_H_4_Cl_2_O_4_)	[Ni(C_13_H_11_N_4_O)_2_](C_8_H_4_Br_2_O_4_)
*M*_r_	772.24	861.14
Crystal system, space group	Triclinic, *P* 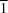	Triclinic, *P* 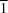
Temperature (K)	123	123
*a*, *b*, *c* (Å)	7.9596 (18), 8.7600 (17), 24.121 (4)	7.8740 (14), 8.9716 (15), 24.233 (4)
α, β, γ (°)	76.138 (8), 81.803 (8), 87.253 (9)	75.040 (9), 82.162 (10), 86.007 (11)
*V* (Å^3^)	1616.0 (6)	1637.3 (5)
*Z*	2	2
Radiation type	Mo *K*α	Mo *K*α
μ (mm^−1^)	0.83	3.10
Crystal size (mm)	0.02 × 0.02 × 0.01	0.02 × 0.02 × 0.01

Data collection		
Diffractometer	Rigaku Saturn724	Rigaku Saturn724
Absorption correction	Multi-scan (*REQAB*; Rigaku, 1998 ▸)	Multi-scan (*REQAB*; Rigaku, 1998 ▸)
*T*_min_, *T*_max_	0.761, 0.848	0.467, 0.538
No. of measured, independent and observed [*F* ^2^ > 2.0σ(*F* ^2^)] reflections	27850, 7359, 6814	28760, 7439, 6665
*R*_int_	0.044	0.050
(sin θ/λ)_max_ (Å^−1^)	0.649	0.648

Refinement		
*R*[*F* ^2^ > 2σ(*F* ^2^)], *wR*(*F* ^2^), *S*	0.053, 0.130, 1.13	0.051, 0.107, 1.14
No. of reflections	7359	7439
No. of parameters	464	464
H-atom treatment	H-atom parameters constrained	H-atom parameters constrained
Δρ_max_, Δρ_min_ (e Å^−3^)	0.57, −0.92	0.72, −0.75

Computer programs: *CrystalClear* and *CrystalStructure* (Rigaku, 2014 ▸), *SIR97* (Altomare *et al.*, 1999 ▸) and *SHELXL2013* (Sheldrick, 2015 ▸).

## supplementary crystallographic information

### (1-Cl) Bis{N-[1-(pyridin-2-yl-κN)ethylidene]pyridine-4-carbohydrazonato-κ2N',O}nickel(II)–2,5-dichloroterephthalic acid (1/1) Crystal data

**Table:**

[Ni(C_13_H_11_N_4_O)_2_](C_8_H_4_Cl_2_O_4_)	*Z* = 2
*M**_r_* = 772.24	*F*(000) = 792.00
Triclinic, *P*1	*D*_x_ = 1.587 Mg m^−^^3^
*a* = 7.9596 (18) Å	Mo *K*α radiation, λ = 0.71075 Å
*b* = 8.7600 (17) Å	Cell parameters from 5812 reflections
*c* = 24.121 (4) Å	θ = 1.8–30.7°
α = 76.138 (8)°	µ = 0.83 mm^−^^1^
β = 81.803 (8)°	*T* = 123 K
γ = 87.253 (9)°	Plate, brown
*V* = 1616.0 (6) Å^3^	0.02 × 0.02 × 0.01 mm

### (1-Cl) Bis{N-[1-(pyridin-2-yl-κN)ethylidene]pyridine-4-carbohydrazonato-κ2N',O}nickel(II)–2,5-dichloroterephthalic acid (1/1) Data collection

**Table:**

Rigaku Saturn724 diffractometer	6814 reflections with *F*^2^ > 2.0σ(*F*^2^)
Detector resolution: 14.222 pixels mm^-1^	*R*_int_ = 0.044
ω scans	θ_max_ = 27.5°, θ_min_ = 3.1°
Absorption correction: multi-scan (*REQAB*; Rigaku, 1998)	*h* = −10→10
*T*_min_ = 0.761, *T*_max_ = 0.848	*k* = −11→11
27850 measured reflections	*l* = −31→31
7359 independent reflections	

### (1-Cl) Bis{N-[1-(pyridin-2-yl-κN)ethylidene]pyridine-4-carbohydrazonato-κ2N',O}nickel(II)–2,5-dichloroterephthalic acid (1/1) Refinement

**Table:**

Refinement on *F*^2^	Secondary atom site location: difference Fourier map
*R*[*F*^2^ > 2σ(*F*^2^)] = 0.053	Hydrogen site location: inferred from neighbouring sites
*wR*(*F*^2^) = 0.130	H-atom parameters constrained
*S* = 1.13	*w* = 1/[σ^2^(*F*_o_^2^) + (0.0612*P*)^2^ + 1.4269*P*] where *P* = (*F*_o_^2^ + 2*F*_c_^2^)/3
7359 reflections	(Δ/σ)_max_ = 0.001
464 parameters	Δρ_max_ = 0.57 e Å^−^^3^
0 restraints	Δρ_min_ = −0.92 e Å^−^^3^
Primary atom site location: structure-invariant direct methods	

### (1-Cl) Bis{N-[1-(pyridin-2-yl-κN)ethylidene]pyridine-4-carbohydrazonato-κ2N',O}nickel(II)–2,5-dichloroterephthalic acid (1/1) Special details

**Table:**

Geometry. ENTER SPECIAL DETAILS OF THE MOLECULAR GEOMETRY
Refinement. Refinement was performed using all reflections. The weighted R-factor (wR) and goodness of fit (S) are based on F^2^. R-factor (gt) are based on F. The threshold expression of F^2^ > 2.0 sigma(F^2^) is used only for calculating R-factor (gt).

### (1-Cl) Bis{N-[1-(pyridin-2-yl-κN)ethylidene]pyridine-4-carbohydrazonato-κ2N',O}nickel(II)–2,5-dichloroterephthalic acid (1/1) Fractional atomic coordinates and isotropic or equivalent isotropic displacement parameters (Å^2^)

**Table:**

	*x*	*y*	*z*	*U*_iso_*/*U*_eq_	
Ni1	0.67086 (4)	0.43172 (3)	0.25224 (2)	0.01854 (10)	
Cl1	0.41155 (8)	−0.35525 (7)	0.03245 (3)	0.02750 (15)	
Cl2	0.79694 (9)	−0.13848 (8)	0.62117 (3)	0.03312 (17)	
O1	0.7541 (2)	0.2053 (2)	0.28890 (7)	0.0231 (4)	
O2	0.4392 (2)	0.3451 (2)	0.24024 (7)	0.0216 (4)	
O3	0.8331 (3)	0.3969 (2)	0.48427 (8)	0.0337 (5)	
O4	1.0219 (3)	0.4091 (2)	0.40572 (9)	0.0391 (5)	
O5	0.7989 (2)	0.0224 (2)	0.09773 (8)	0.0285 (4)	
O6	0.9030 (3)	0.1760 (3)	0.01231 (8)	0.0330 (4)	
N1	0.5819 (3)	0.6608 (2)	0.25437 (9)	0.0197 (4)	
N2	0.6092 (3)	0.4157 (2)	0.33633 (8)	0.0188 (4)	
N3	0.6286 (3)	0.2722 (2)	0.37388 (9)	0.0212 (4)	
N4	0.7897 (3)	−0.2955 (3)	0.43917 (10)	0.0298 (5)	
N5	0.9154 (3)	0.5276 (3)	0.22513 (9)	0.0210 (4)	
N6	0.7008 (3)	0.4439 (2)	0.16820 (8)	0.0184 (4)	
N7	0.5775 (3)	0.3818 (2)	0.14568 (8)	0.0198 (4)	
N8	0.0405 (3)	0.1303 (3)	0.13524 (9)	0.0236 (4)	
C1	0.5204 (3)	0.6749 (3)	0.30831 (10)	0.0187 (5)	
C2	0.4620 (3)	0.8194 (3)	0.31865 (11)	0.0216 (5)	
C3	0.4758 (3)	0.9523 (3)	0.27352 (11)	0.0253 (5)	
C4	0.5456 (3)	0.9378 (3)	0.21886 (11)	0.0243 (5)	
C5	0.5948 (3)	0.7898 (3)	0.21134 (10)	0.0224 (5)	
C6	0.5269 (3)	0.5291 (3)	0.35434 (10)	0.0197 (5)	
C7	0.4446 (4)	0.5154 (3)	0.41466 (11)	0.0292 (6)	
C8	0.7073 (3)	0.1748 (3)	0.34310 (10)	0.0199 (5)	
C9	0.7388 (3)	0.0127 (3)	0.37742 (10)	0.0214 (5)	
C10	0.6540 (4)	−0.0432 (3)	0.43257 (11)	0.0277 (6)	
C11	0.6816 (4)	−0.1960 (3)	0.46184 (12)	0.0322 (6)	
C12	0.8510 (4)	−0.0892 (3)	0.35420 (11)	0.0256 (5)	
C13	0.8734 (4)	−0.2401 (3)	0.38644 (11)	0.0285 (6)	
C14	0.9496 (3)	0.5711 (3)	0.16687 (10)	0.0209 (5)	
C15	1.0827 (3)	0.6692 (3)	0.13930 (12)	0.0292 (6)	
C16	1.1852 (4)	0.7223 (4)	0.17243 (13)	0.0368 (7)	
C17	1.1539 (4)	0.6737 (4)	0.23198 (13)	0.0364 (7)	
C18	1.0174 (3)	0.5764 (3)	0.25663 (12)	0.0272 (5)	
C19	0.8334 (3)	0.5091 (3)	0.13497 (10)	0.0195 (5)	
C20	0.8694 (3)	0.5198 (3)	0.07165 (10)	0.0250 (5)	
C21	0.4492 (3)	0.3366 (3)	0.18769 (10)	0.0195 (5)	
C22	0.3055 (3)	0.2660 (3)	0.16921 (10)	0.0202 (5)	
C23	0.3146 (3)	0.2441 (3)	0.11342 (11)	0.0243 (5)	
C24	0.1815 (3)	0.1762 (3)	0.09809 (11)	0.0266 (5)	
C25	0.1580 (3)	0.2183 (3)	0.20755 (10)	0.0208 (5)	
C26	0.0296 (3)	0.1524 (3)	0.18879 (11)	0.0234 (5)	
C27	0.7947 (3)	0.0902 (3)	0.04348 (11)	0.0232 (5)	
C28	0.6401 (3)	0.0472 (3)	0.02141 (10)	0.0215 (5)	
C29	0.5893 (3)	−0.1087 (3)	0.03644 (10)	0.0221 (5)	
C30	0.4537 (3)	−0.1554 (3)	0.01532 (10)	0.0207 (5)	
C31	0.9135 (3)	−0.0673 (3)	0.55392 (11)	0.0236 (5)	
C32	0.8909 (3)	0.0907 (3)	0.52835 (11)	0.0242 (5)	
C33	0.9751 (3)	0.1619 (3)	0.47446 (11)	0.0226 (5)	
C34	0.9472 (3)	0.3356 (3)	0.45046 (11)	0.0249 (5)	
H1	0.41316	0.82673	0.35624	0.0260*	
H2	0.43799	1.05195	0.27992	0.0303*	
H3	0.55916	1.02732	0.1874	0.0291*	
H4	0.64003	0.7793	0.17378	0.0269*	
H5B	0.51437	0.44762	0.44126	0.0351*	
H6C	0.3319	0.46964	0.41959	0.0351*	
H7A	0.4332	0.61998	0.42288	0.0351*	
H8	0.5774	0.02358	0.45005	0.0333*	
H9	0.62211	−0.23263	0.49945	0.0386*	
H10	0.91159	−0.05553	0.31661	0.0307*	
H11	0.95218	−0.30803	0.37036	0.0342*	
H12	1.10347	0.69948	0.09841	0.0351*	
H13	1.27605	0.79128	0.1545	0.0442*	
H14	1.22456	0.70646	0.25544	0.0437*	
H15	0.99564	0.54319	0.29744	0.0326*	
H16C	0.96065	0.44528	0.06411	0.0299*	
H17A	0.90443	0.62684	0.05154	0.0299*	
H18B	0.76681	0.49436	0.05776	0.0299*	
H19	0.41227	0.27593	0.08615	0.0291*	
H20	0.18943	0.16126	0.06006	0.0319*	
H21	0.14673	0.23126	0.24591	0.0250*	
H22	−0.07078	0.12132	0.21482	0.0280*	
H23	0.64945	−0.18442	0.06181	0.0265*	
H24	0.81501	0.15245	0.54843	0.0290*	
H25	0.82374	0.49396	0.4703	0.0404*	
H26	0.882	0.05571	0.10908	0.0341*	

### (1-Cl) Bis{N-[1-(pyridin-2-yl-κN)ethylidene]pyridine-4-carbohydrazonato-κ2N',O}nickel(II)–2,5-dichloroterephthalic acid (1/1) Atomic displacement parameters (Å^2^)

**Table:**

	*U*^11^	*U*^22^	*U*^33^	*U*^12^	*U*^13^	*U*^23^
Ni1	0.02329 (18)	0.01591 (17)	0.01524 (16)	0.00197 (12)	−0.00110 (12)	−0.00266 (12)
Cl1	0.0335 (3)	0.0171 (3)	0.0299 (3)	−0.0019 (2)	−0.0009 (3)	−0.0035 (2)
Cl2	0.0421 (4)	0.0239 (3)	0.0274 (3)	0.0045 (3)	0.0084 (3)	−0.0030 (3)
O1	0.0305 (10)	0.0197 (9)	0.0174 (8)	0.0048 (7)	−0.0001 (7)	−0.0036 (7)
O2	0.0253 (9)	0.0213 (9)	0.0182 (8)	−0.0013 (7)	−0.0008 (7)	−0.0058 (7)
O3	0.0475 (12)	0.0179 (9)	0.0294 (10)	0.0098 (9)	0.0061 (9)	−0.0019 (8)
O4	0.0489 (13)	0.0232 (10)	0.0352 (11)	0.0071 (9)	0.0109 (10)	0.0015 (9)
O5	0.0267 (10)	0.0383 (11)	0.0188 (9)	−0.0092 (8)	−0.0055 (7)	−0.0004 (8)
O6	0.0336 (11)	0.0384 (11)	0.0238 (10)	−0.0130 (9)	−0.0042 (8)	0.0018 (8)
N1	0.0221 (10)	0.0177 (10)	0.0185 (10)	0.0029 (8)	−0.0025 (8)	−0.0036 (8)
N2	0.0218 (10)	0.0165 (10)	0.0170 (9)	0.0001 (8)	−0.0014 (8)	−0.0024 (8)
N3	0.0280 (11)	0.0166 (10)	0.0171 (10)	0.0016 (8)	−0.0025 (8)	−0.0008 (8)
N4	0.0436 (14)	0.0178 (11)	0.0263 (12)	0.0037 (10)	−0.0066 (10)	−0.0019 (9)
N5	0.0213 (10)	0.0217 (10)	0.0198 (10)	0.0039 (8)	−0.0032 (8)	−0.0050 (8)
N6	0.0214 (10)	0.0158 (10)	0.0175 (9)	0.0018 (8)	−0.0030 (8)	−0.0030 (8)
N7	0.0226 (10)	0.0201 (10)	0.0167 (9)	−0.0011 (8)	−0.0024 (8)	−0.0041 (8)
N8	0.0235 (11)	0.0242 (11)	0.0214 (10)	−0.0001 (9)	−0.0030 (8)	−0.0023 (9)
C1	0.0165 (11)	0.0194 (11)	0.0195 (11)	−0.0006 (9)	−0.0028 (9)	−0.0033 (9)
C2	0.0271 (13)	0.0169 (11)	0.0199 (12)	0.0008 (9)	−0.0006 (9)	−0.0039 (9)
C3	0.0279 (13)	0.0200 (12)	0.0288 (13)	0.0041 (10)	−0.0052 (10)	−0.0074 (10)
C4	0.0295 (13)	0.0168 (12)	0.0230 (12)	0.0028 (10)	−0.0020 (10)	0.0008 (10)
C5	0.0233 (12)	0.0228 (12)	0.0179 (11)	0.0028 (10)	−0.0017 (9)	0.0002 (9)
C6	0.0210 (12)	0.0186 (12)	0.0183 (11)	−0.0004 (9)	−0.0001 (9)	−0.0032 (9)
C7	0.0407 (16)	0.0206 (13)	0.0214 (13)	0.0037 (11)	0.0061 (11)	−0.0020 (10)
C8	0.0220 (12)	0.0180 (12)	0.0184 (11)	0.0013 (9)	−0.0036 (9)	−0.0014 (9)
C9	0.0263 (13)	0.0179 (12)	0.0193 (12)	0.0020 (10)	−0.0051 (9)	−0.0025 (9)
C10	0.0352 (15)	0.0224 (13)	0.0220 (12)	0.0033 (11)	0.0016 (11)	−0.0022 (10)
C11	0.0437 (17)	0.0218 (13)	0.0241 (13)	0.0044 (12)	0.0028 (12)	0.0028 (11)
C12	0.0323 (14)	0.0221 (13)	0.0216 (12)	0.0038 (10)	−0.0032 (10)	−0.0049 (10)
C13	0.0389 (15)	0.0209 (13)	0.0257 (13)	0.0081 (11)	−0.0044 (11)	−0.0071 (10)
C14	0.0196 (12)	0.0210 (12)	0.0212 (12)	0.0054 (9)	−0.0015 (9)	−0.0050 (9)
C15	0.0244 (13)	0.0356 (15)	0.0259 (13)	−0.0007 (11)	−0.0022 (10)	−0.0043 (11)
C16	0.0245 (14)	0.0495 (19)	0.0356 (16)	−0.0112 (13)	−0.0011 (12)	−0.0080 (14)
C17	0.0266 (14)	0.0505 (19)	0.0354 (16)	−0.0052 (13)	−0.0077 (12)	−0.0136 (14)
C18	0.0252 (13)	0.0338 (14)	0.0232 (13)	0.0030 (11)	−0.0050 (10)	−0.0078 (11)
C19	0.0210 (12)	0.0171 (11)	0.0176 (11)	0.0029 (9)	−0.0006 (9)	−0.0004 (9)
C20	0.0284 (13)	0.0268 (13)	0.0176 (12)	−0.0020 (10)	0.0019 (10)	−0.0037 (10)
C21	0.0250 (12)	0.0138 (11)	0.0180 (11)	0.0051 (9)	−0.0034 (9)	−0.0015 (9)
C22	0.0244 (12)	0.0131 (11)	0.0216 (12)	0.0053 (9)	−0.0033 (9)	−0.0021 (9)
C23	0.0233 (12)	0.0298 (14)	0.0192 (12)	0.0008 (10)	0.0007 (9)	−0.0069 (10)
C24	0.0276 (13)	0.0346 (14)	0.0184 (12)	−0.0004 (11)	−0.0010 (10)	−0.0092 (11)
C25	0.0231 (12)	0.0199 (12)	0.0184 (11)	0.0019 (9)	−0.0007 (9)	−0.0039 (9)
C26	0.0247 (13)	0.0235 (13)	0.0206 (12)	0.0024 (10)	−0.0015 (9)	−0.0040 (10)
C27	0.0269 (13)	0.0217 (12)	0.0202 (12)	−0.0004 (10)	−0.0016 (10)	−0.0044 (10)
C28	0.0228 (12)	0.0232 (12)	0.0176 (11)	0.0002 (10)	0.0004 (9)	−0.0048 (9)
C29	0.0273 (13)	0.0187 (12)	0.0187 (11)	0.0031 (10)	−0.0027 (9)	−0.0020 (9)
C30	0.0264 (12)	0.0182 (11)	0.0168 (11)	0.0001 (9)	−0.0012 (9)	−0.0039 (9)
C31	0.0267 (13)	0.0224 (12)	0.0205 (12)	0.0024 (10)	−0.0006 (10)	−0.0051 (10)
C32	0.0268 (13)	0.0212 (12)	0.0254 (13)	0.0034 (10)	−0.0019 (10)	−0.0089 (10)
C33	0.0246 (13)	0.0197 (12)	0.0242 (12)	0.0013 (10)	−0.0051 (10)	−0.0054 (10)
C34	0.0305 (14)	0.0179 (12)	0.0255 (13)	0.0041 (10)	−0.0029 (10)	−0.0050 (10)

### (1-Cl) Bis{N-[1-(pyridin-2-yl-κN)ethylidene]pyridine-4-carbohydrazonato-κ2N',O}nickel(II)–2,5-dichloroterephthalic acid (1/1) Geometric parameters (Å, º)

**Table:**

Ni1—O1	2.0870 (17)	C16—C17	1.386 (4)
Ni1—O2	2.1099 (19)	C17—C18	1.386 (4)
Ni1—N1	2.106 (2)	C19—C20	1.494 (3)
Ni1—N2	1.990 (2)	C21—C22	1.495 (4)
Ni1—N5	2.110 (2)	C22—C23	1.396 (4)
Ni1—N6	1.985 (2)	C22—C25	1.400 (3)
Cl1—C30	1.738 (3)	C23—C24	1.374 (4)
Cl2—C31	1.739 (2)	C25—C26	1.377 (4)
O1—C8	1.274 (3)	C27—C28	1.506 (4)
O2—C21	1.278 (3)	C28—C29	1.391 (4)
C34—O3	1.316 (3)	C28—C30^i^	1.400 (3)
C34—O4	1.206 (3)	C29—C30	1.375 (4)
C27—O5	1.306 (3)	C31—C32	1.388 (3)
C27—O6	1.216 (3)	C31—C33^ii^	1.404 (4)
N1—C1	1.355 (3)	C32—C33	1.390 (3)
N1—C5	1.335 (3)	C33—C34	1.510 (3)
N2—N3	1.379 (3)	O3—H25	0.840
N2—C6	1.292 (3)	O5—H26	0.840
N3—C8	1.338 (3)	C2—H1	0.950
N4—C11	1.350 (4)	C3—H2	0.950
N4—C13	1.338 (3)	C4—H3	0.950
N5—C14	1.356 (3)	C5—H4	0.950
N5—C18	1.336 (4)	C7—H5B	0.980
N6—N7	1.378 (3)	C7—H6C	0.980
N6—C19	1.296 (3)	C7—H7A	0.980
N7—C21	1.333 (3)	C10—H8	0.950
N8—C24	1.345 (3)	C11—H9	0.950
N8—C26	1.342 (4)	C12—H10	0.950
C1—C2	1.393 (4)	C13—H11	0.950
C1—C6	1.482 (3)	C15—H12	0.950
C2—C3	1.387 (3)	C16—H13	0.950
C3—C4	1.389 (4)	C17—H14	0.950
C4—C5	1.382 (4)	C18—H15	0.950
C6—C7	1.488 (3)	C20—H16C	0.980
C8—C9	1.492 (3)	C20—H17A	0.980
C9—C10	1.389 (3)	C20—H18B	0.980
C9—C12	1.389 (4)	C23—H19	0.950
C10—C11	1.379 (4)	C24—H20	0.950
C12—C13	1.382 (3)	C25—H21	0.950
C14—C15	1.384 (3)	C26—H22	0.950
C14—C19	1.481 (4)	C29—H23	0.950
C15—C16	1.386 (5)	C32—H24	0.950
			
O1—Ni1—O2	91.34 (7)	N8—C26—C25	122.6 (2)
O1—Ni1—N1	154.21 (8)	O5—C27—O6	125.6 (3)
O1—Ni1—N2	77.13 (8)	O5—C27—C28	112.6 (2)
O1—Ni1—N5	95.75 (8)	O6—C27—C28	121.8 (2)
O1—Ni1—N6	104.55 (8)	C27—C28—C29	119.0 (2)
O2—Ni1—N1	96.67 (8)	C27—C28—C30^i^	123.5 (2)
O2—Ni1—N2	95.71 (8)	C29—C28—C30^i^	117.5 (2)
O2—Ni1—N5	154.86 (8)	C28—C29—C30	121.3 (2)
O2—Ni1—N6	77.12 (8)	Cl1—C30—C28^i^	121.3 (2)
N1—Ni1—N2	77.70 (8)	Cl1—C30—C29	117.39 (18)
N1—Ni1—N5	87.32 (8)	C28^i^—C30—C29	121.2 (2)
N1—Ni1—N6	101.13 (8)	Cl2—C31—C32	116.7 (2)
N2—Ni1—N5	109.38 (9)	Cl2—C31—C33^ii^	123.01 (18)
N2—Ni1—N6	172.61 (9)	C32—C31—C33^ii^	120.3 (2)
N5—Ni1—N6	77.75 (9)	C31—C32—C33	122.4 (2)
Ni1—O1—C8	108.99 (15)	C31^ii^—C33—C32	117.2 (2)
Ni1—O2—C21	108.26 (15)	C31^ii^—C33—C34	123.7 (2)
Ni1—N1—C1	112.70 (15)	C32—C33—C34	119.1 (2)
Ni1—N1—C5	128.03 (18)	O3—C34—O4	124.2 (2)
C1—N1—C5	118.9 (2)	O3—C34—C33	112.1 (2)
Ni1—N2—N3	118.43 (16)	O4—C34—C33	123.7 (2)
Ni1—N2—C6	120.19 (15)	C34—O3—H25	109.478
N3—N2—C6	120.36 (19)	C27—O5—H26	109.477
N2—N3—C8	107.53 (19)	C1—C2—H1	120.325
C11—N4—C13	117.4 (2)	C3—C2—H1	120.322
Ni1—N5—C14	111.70 (17)	C2—C3—H2	120.450
Ni1—N5—C18	128.02 (17)	C4—C3—H2	120.447
C14—N5—C18	118.7 (2)	C3—C4—H3	120.816
Ni1—N6—N7	118.72 (13)	C5—C4—H3	120.812
Ni1—N6—C19	120.82 (19)	N1—C5—H4	118.447
N7—N6—C19	120.5 (2)	C4—C5—H4	118.448
N6—N7—C21	108.0 (2)	C6—C7—H5B	109.470
C24—N8—C26	118.8 (2)	C6—C7—H6C	109.478
N1—C1—C2	121.0 (2)	C6—C7—H7A	109.475
N1—C1—C6	115.3 (2)	H5B—C7—H6C	109.469
C2—C1—C6	123.6 (2)	H5B—C7—H7A	109.464
C1—C2—C3	119.4 (2)	H6C—C7—H7A	109.472
C2—C3—C4	119.1 (2)	C9—C10—H8	120.135
C3—C4—C5	118.4 (2)	C11—C10—H8	120.151
N1—C5—C4	123.1 (2)	N4—C11—H9	118.708
N2—C6—C1	113.0 (2)	C10—C11—H9	118.699
N2—C6—C7	123.9 (2)	C9—C12—H10	120.404
C1—C6—C7	123.1 (2)	C13—C12—H10	120.405
O1—C8—N3	127.3 (2)	N4—C13—H11	118.362
O1—C8—C9	118.1 (2)	C12—C13—H11	118.358
N3—C8—C9	114.6 (2)	C14—C15—H12	120.607
C8—C9—C10	121.6 (2)	C16—C15—H12	120.613
C8—C9—C12	120.6 (2)	C15—C16—H13	120.324
C10—C9—C12	117.8 (2)	C17—C16—H13	120.330
C9—C10—C11	119.7 (3)	C16—C17—H14	120.669
N4—C11—C10	122.6 (2)	C18—C17—H14	120.655
C9—C12—C13	119.2 (2)	N5—C18—H15	118.782
N4—C13—C12	123.3 (3)	C17—C18—H15	118.778
N5—C14—C15	122.0 (3)	C19—C20—H16C	109.476
N5—C14—C19	115.6 (2)	C19—C20—H17A	109.482
C15—C14—C19	122.5 (2)	C19—C20—H18B	109.472
C14—C15—C16	118.8 (3)	H16C—C20—H17A	109.466
C15—C16—C17	119.3 (3)	H16C—C20—H18B	109.468
C16—C17—C18	118.7 (3)	H17A—C20—H18B	109.463
N5—C18—C17	122.4 (3)	C22—C23—H19	120.140
N6—C19—C14	112.5 (2)	C24—C23—H19	120.125
N6—C19—C20	124.7 (2)	N8—C24—H20	118.975
C14—C19—C20	122.8 (2)	C23—C24—H20	118.982
O2—C21—N7	127.5 (2)	C22—C25—H21	120.510
O2—C21—C22	118.7 (2)	C26—C25—H21	120.521
N7—C21—C22	113.8 (2)	N8—C26—H22	118.675
C21—C22—C23	121.1 (2)	C25—C26—H22	118.677
C21—C22—C25	121.0 (2)	C28—C29—H23	119.339
C23—C22—C25	117.8 (2)	C30—C29—H23	119.342
C22—C23—C24	119.7 (2)	C31—C32—H24	118.785
N8—C24—C23	122.0 (3)	C33—C32—H24	118.786
C22—C25—C26	119.0 (2)		
			
O1—Ni1—O2—C21	−99.87 (11)	Ni1—N6—N7—C21	5.8 (2)
O2—Ni1—O1—C8	−89.54 (12)	Ni1—N6—C19—C14	−0.9 (3)
O1—Ni1—N1—C1	−17.2 (3)	Ni1—N6—C19—C20	178.20 (13)
O1—Ni1—N1—C5	155.73 (14)	N7—N6—C19—C14	178.99 (17)
N1—Ni1—O1—C8	18.9 (3)	N7—N6—C19—C20	−1.9 (3)
O1—Ni1—N2—N3	−7.52 (13)	C19—N6—N7—C21	−174.06 (19)
O1—Ni1—N2—C6	−176.01 (17)	N6—N7—C21—O2	−1.1 (3)
N2—Ni1—O1—C8	6.01 (12)	N6—N7—C21—C22	−179.84 (16)
O1—Ni1—N5—C14	114.34 (13)	C24—N8—C26—C25	−0.9 (3)
O1—Ni1—N5—C18	−80.17 (17)	C26—N8—C24—C23	0.4 (4)
N5—Ni1—O1—C8	114.62 (13)	N1—C1—C2—C3	−3.6 (4)
O1—Ni1—N6—N7	82.01 (14)	N1—C1—C6—N2	7.8 (3)
O1—Ni1—N6—C19	−98.13 (14)	N1—C1—C6—C7	−171.4 (2)
N6—Ni1—O1—C8	−166.58 (12)	C2—C1—C6—N2	−169.9 (2)
O2—Ni1—N1—C1	90.00 (14)	C2—C1—C6—C7	10.9 (4)
O2—Ni1—N1—C5	−97.03 (17)	C6—C1—C2—C3	173.9 (2)
N1—Ni1—O2—C21	104.69 (11)	C1—C2—C3—C4	0.9 (4)
O2—Ni1—N2—N3	82.55 (14)	C2—C3—C4—C5	1.6 (4)
O2—Ni1—N2—C6	−85.94 (16)	C3—C4—C5—N1	−1.5 (4)
N2—Ni1—O2—C21	−177.07 (11)	O1—C8—C9—C10	−162.4 (2)
O2—Ni1—N5—C14	8.7 (3)	O1—C8—C9—C12	15.5 (4)
O2—Ni1—N5—C18	174.18 (12)	N3—C8—C9—C10	16.5 (4)
N5—Ni1—O2—C21	6.7 (2)	N3—C8—C9—C12	−165.5 (2)
O2—Ni1—N6—N7	−6.01 (12)	C8—C9—C10—C11	177.2 (2)
O2—Ni1—N6—C19	173.85 (16)	C8—C9—C12—C13	−178.0 (2)
N6—Ni1—O2—C21	4.75 (10)	C10—C9—C12—C13	0.0 (4)
N1—Ni1—N2—N3	178.16 (16)	C12—C9—C10—C11	−0.8 (4)
N1—Ni1—N2—C6	9.67 (15)	C9—C10—C11—N4	0.4 (5)
N2—Ni1—N1—C1	−4.42 (13)	C9—C12—C13—N4	1.2 (5)
N2—Ni1—N1—C5	168.55 (19)	N5—C14—C15—C16	0.9 (4)
N1—Ni1—N5—C14	−91.35 (14)	N5—C14—C19—N6	10.7 (3)
N1—Ni1—N5—C18	74.15 (17)	N5—C14—C19—C20	−168.44 (19)
N5—Ni1—N1—C1	−114.92 (14)	C15—C14—C19—N6	−168.3 (2)
N5—Ni1—N1—C5	58.05 (18)	C15—C14—C19—C20	12.6 (4)
N1—Ni1—N6—N7	−100.40 (14)	C19—C14—C15—C16	179.8 (2)
N1—Ni1—N6—C19	79.46 (15)	C14—C15—C16—C17	1.2 (4)
N6—Ni1—N1—C1	168.12 (13)	C15—C16—C17—C18	−1.7 (5)
N6—Ni1—N1—C5	−18.91 (19)	C16—C17—C18—N5	0.1 (4)
N2—Ni1—N5—C14	−167.31 (12)	O2—C21—C22—C23	−175.62 (18)
N2—Ni1—N5—C18	−1.82 (19)	O2—C21—C22—C25	3.7 (3)
N5—Ni1—N2—N3	−99.16 (15)	N7—C21—C22—C23	3.2 (3)
N5—Ni1—N2—C6	92.36 (16)	N7—C21—C22—C25	−177.40 (18)
N5—Ni1—N6—N7	174.84 (15)	C21—C22—C23—C24	178.79 (19)
N5—Ni1—N6—C19	−5.30 (14)	C21—C22—C25—C26	−179.32 (18)
N6—Ni1—N5—C14	10.66 (13)	C23—C22—C25—C26	0.0 (3)
N6—Ni1—N5—C18	176.15 (18)	C25—C22—C23—C24	−0.6 (3)
Ni1—O1—C8—N3	−4.5 (3)	C22—C23—C24—N8	0.4 (4)
Ni1—O1—C8—C9	174.20 (14)	C22—C25—C26—N8	0.7 (4)
Ni1—O2—C21—N7	−3.5 (3)	O5—C27—C28—C29	−43.4 (3)
Ni1—O2—C21—C22	175.16 (13)	O5—C27—C28—C30^i^	138.8 (2)
Ni1—N1—C1—C2	177.44 (15)	O6—C27—C28—C29	134.9 (2)
Ni1—N1—C1—C6	−0.3 (2)	O6—C27—C28—C30^i^	−43.0 (4)
Ni1—N1—C5—C4	−173.77 (15)	C27—C28—C29—C30	−177.0 (2)
C1—N1—C5—C4	−1.2 (4)	C27—C28—C30^i^—Cl1^i^	−7.1 (3)
C5—N1—C1—C2	3.8 (3)	C27—C28—C30^i^—C29^i^	176.9 (2)
C5—N1—C1—C6	−173.9 (2)	C29—C28—C30^i^—Cl1^i^	175.04 (18)
Ni1—N2—N3—C8	7.2 (2)	C29—C28—C30^i^—C29^i^	−1.0 (3)
Ni1—N2—C6—C1	−12.4 (3)	C30^i^—C28—C29—C30	1.0 (3)
Ni1—N2—C6—C7	166.82 (15)	C28—C29—C30—Cl1	175.15 (19)
N3—N2—C6—C1	179.38 (19)	C28—C29—C30—C28^i^	−1.0 (4)
N3—N2—C6—C7	−1.4 (4)	Cl2—C31—C32—C33	−179.38 (18)
C6—N2—N3—C8	175.6 (2)	Cl2—C31—C33^ii^—C32^ii^	179.35 (18)
N2—N3—C8—O1	−1.3 (4)	Cl2—C31—C33^ii^—C34^ii^	1.3 (4)
N2—N3—C8—C9	179.92 (18)	C32—C31—C33^ii^—C32^ii^	−0.1 (4)
C11—N4—C13—C12	−1.7 (4)	C32—C31—C33^ii^—C34^ii^	−178.2 (2)
C13—N4—C11—C10	0.9 (4)	C33^ii^—C31—C32—C33	0.1 (4)
Ni1—N5—C14—C15	164.49 (15)	C31—C32—C33—C31^ii^	−0.1 (4)
Ni1—N5—C14—C19	−14.5 (2)	C31—C32—C33—C34	−178.3 (2)
Ni1—N5—C18—C17	−162.65 (15)	C31^ii^—C33—C34—O3	178.1 (2)
C14—N5—C18—C17	2.0 (4)	C31^ii^—C33—C34—O4	−2.4 (5)
C18—N5—C14—C15	−2.5 (3)	C32—C33—C34—O3	−3.9 (4)
C18—N5—C14—C19	178.5 (2)	C32—C33—C34—O4	175.6 (3)

Symmetry codes: (i) −*x*+1, −*y*, −*z*; (ii) −*x*+2, −*y*, −*z*+1.

### (1-Cl) Bis{N-[1-(pyridin-2-yl-κN)ethylidene]pyridine-4-carbohydrazonato-κ2N',O}nickel(II)–2,5-dichloroterephthalic acid (1/1) Hydrogen-bond geometry (Å, º)

**Table:**

*D*—H···*A*	*D*—H	H···*A*	*D*···*A*	*D*—H···*A*
O3—H25···N4^iii^	0.84	1.84	2.679 (3)	177
O5—H26···N8^iv^	0.84	1.71	2.547 (3)	176

Symmetry codes: (iii) *x*, *y*+1, *z*; (iv) *x*+1, *y*, *z*.

### (1-Br) Bis{N-[1-(pyridin-2-yl-κN)ethylidene]pyridine-4-carbohydrazonato-κ2N',O}nickel(II)–2,5-dibromoterephthalic acid (1/1) Crystal data

**Table:**

[Ni(C_13_H_11_N_4_O)_2_](C_8_H_4_Br_2_O_4_)	*Z* = 2
*M**_r_* = 861.14	*F*(000) = 864.00
Triclinic, *P*1	*D*_x_ = 1.747 Mg m^−^^3^
*a* = 7.8740 (14) Å	Mo *K*α radiation, λ = 0.71075 Å
*b* = 8.9716 (15) Å	Cell parameters from 6236 reflections
*c* = 24.233 (4) Å	θ = 1.8–30.8°
α = 75.040 (9)°	µ = 3.10 mm^−^^1^
β = 82.162 (10)°	*T* = 123 K
γ = 86.007 (11)°	Plate, brown
*V* = 1637.3 (5) Å^3^	0.02 × 0.02 × 0.01 mm

### (1-Br) Bis{N-[1-(pyridin-2-yl-κN)ethylidene]pyridine-4-carbohydrazonato-κ2N',O}nickel(II)–2,5-dibromoterephthalic acid (1/1) Data collection

**Table:**

Rigaku Saturn724 diffractometer	6665 reflections with *F*^2^ > 2.0σ(*F*^2^)
Detector resolution: 14.222 pixels mm^-1^	*R*_int_ = 0.050
ω scans	θ_max_ = 27.4°, θ_min_ = 3.1°
Absorption correction: multi-scan (*REQAB*; Rigaku, 1998)	*h* = −10→10
*T*_min_ = 0.467, *T*_max_ = 0.538	*k* = −11→11
28760 measured reflections	*l* = −31→31
7439 independent reflections	

### (1-Br) Bis{N-[1-(pyridin-2-yl-κN)ethylidene]pyridine-4-carbohydrazonato-κ2N',O}nickel(II)–2,5-dibromoterephthalic acid (1/1) Refinement

**Table:**

Refinement on *F*^2^	Secondary atom site location: difference Fourier map
*R*[*F*^2^ > 2σ(*F*^2^)] = 0.051	Hydrogen site location: inferred from neighbouring sites
*wR*(*F*^2^) = 0.107	H-atom parameters constrained
*S* = 1.14	*w* = 1/[σ^2^(*F*_o_^2^) + (0.036*P*)^2^ + 2.3422*P*] where *P* = (*F*_o_^2^ + 2*F*_c_^2^)/3
7439 reflections	(Δ/σ)_max_ = 0.001
464 parameters	Δρ_max_ = 0.72 e Å^−^^3^
0 restraints	Δρ_min_ = −0.75 e Å^−^^3^
Primary atom site location: structure-invariant direct methods	

### (1-Br) Bis{N-[1-(pyridin-2-yl-κN)ethylidene]pyridine-4-carbohydrazonato-κ2N',O}nickel(II)–2,5-dibromoterephthalic acid (1/1) Special details

**Table:**

Geometry. ENTER SPECIAL DETAILS OF THE MOLECULAR GEOMETRY
Refinement. Refinement was performed using all reflections. The weighted R-factor (wR) and goodness of fit (S) are based on F^2^. R-factor (gt) are based on F. The threshold expression of F^2^ > 2.0 sigma(F^2^) is used only for calculating R-factor (gt).

### (1-Br) Bis{N-[1-(pyridin-2-yl-κN)ethylidene]pyridine-4-carbohydrazonato-κ2N',O}nickel(II)–2,5-dibromoterephthalic acid (1/1) Fractional atomic coordinates and isotropic or equivalent isotropic displacement parameters (Å^2^)

**Table:**

	*x*	*y*	*z*	*U*_iso_*/*U*_eq_	
Br1	0.08134 (5)	0.36721 (4)	0.45974 (2)	0.03504 (11)	
Br2	0.71262 (5)	0.14742 (4)	0.87301 (2)	0.02901 (11)	
Ni1	0.17274 (6)	0.41976 (5)	0.75363 (2)	0.02102 (11)	
O1	0.2587 (3)	0.1969 (3)	0.79096 (10)	0.0250 (5)	
O2	−0.0585 (3)	0.3334 (3)	0.74089 (9)	0.0236 (5)	
O3	0.3310 (4)	0.3923 (3)	0.98699 (11)	0.0321 (6)	
O4	0.5246 (4)	0.4085 (3)	0.90949 (12)	0.0394 (7)	
O5	0.3029 (3)	0.0259 (3)	0.59558 (10)	0.0335 (6)	
O6	0.4060 (4)	0.1768 (3)	0.50971 (11)	0.0392 (7)	
N1	0.0800 (4)	0.6459 (3)	0.75401 (12)	0.0219 (6)	
N2	0.1146 (4)	0.4059 (3)	0.83712 (11)	0.0198 (6)	
N3	0.1365 (4)	0.2643 (3)	0.87523 (11)	0.0206 (6)	
N4	0.2905 (4)	−0.3001 (3)	0.94192 (13)	0.0280 (7)	
N5	0.4171 (4)	0.5170 (3)	0.72801 (12)	0.0230 (6)	
N6	0.2067 (4)	0.4314 (3)	0.67004 (11)	0.0207 (6)	
N7	0.0867 (4)	0.3661 (3)	0.64776 (11)	0.0226 (6)	
N8	−0.4536 (4)	0.1265 (3)	0.63430 (12)	0.0262 (6)	
C1	0.0264 (4)	0.6627 (4)	0.80766 (14)	0.0201 (7)	
C2	−0.0243 (5)	0.8064 (4)	0.81732 (15)	0.0250 (7)	
C3	−0.0113 (5)	0.9368 (4)	0.77106 (15)	0.0278 (8)	
C4	0.0499 (5)	0.9204 (4)	0.71676 (15)	0.0281 (8)	
C5	0.0911 (5)	0.7722 (4)	0.70999 (15)	0.0264 (8)	
C6	0.0339 (4)	0.5190 (4)	0.85436 (14)	0.0210 (7)	
C7	−0.0436 (5)	0.5096 (4)	0.91452 (14)	0.0275 (8)	
C8	0.2134 (4)	0.1678 (4)	0.84499 (14)	0.0211 (7)	
C9	0.2437 (4)	0.0059 (4)	0.87960 (14)	0.0210 (7)	
C10	0.1563 (5)	−0.0480 (4)	0.93437 (15)	0.0277 (8)	
C11	0.1833 (5)	−0.2009 (4)	0.96349 (16)	0.0308 (8)	
C12	0.3547 (5)	−0.0961 (4)	0.85712 (15)	0.0260 (7)	
C13	0.3756 (5)	−0.2461 (4)	0.88957 (15)	0.0275 (8)	
C14	0.4490 (4)	0.5644 (4)	0.66952 (14)	0.0234 (7)	
C15	0.5748 (5)	0.6703 (4)	0.64261 (16)	0.0303 (8)	
C16	0.6693 (5)	0.7273 (5)	0.67645 (18)	0.0394 (10)	
C17	0.6416 (5)	0.6749 (5)	0.73531 (17)	0.0366 (9)	
C18	0.5135 (5)	0.5698 (4)	0.75984 (16)	0.0291 (8)	
C19	0.3388 (4)	0.4983 (4)	0.63754 (14)	0.0221 (7)	
C20	0.3813 (5)	0.5089 (4)	0.57493 (14)	0.0291 (8)	
C21	−0.0439 (5)	0.3231 (4)	0.68878 (14)	0.0224 (7)	
C22	−0.1881 (4)	0.2546 (4)	0.66969 (14)	0.0216 (7)	
C23	−0.1743 (5)	0.2322 (4)	0.61435 (15)	0.0266 (8)	
C24	−0.3091 (5)	0.1685 (4)	0.59875 (15)	0.0298 (8)	
C25	−0.3379 (5)	0.2111 (4)	0.70672 (14)	0.0233 (7)	
C26	−0.4671 (5)	0.1476 (4)	0.68768 (15)	0.0263 (7)	
C27	0.2984 (5)	0.0920 (4)	0.54129 (15)	0.0285 (8)	
C28	0.1417 (5)	0.0496 (4)	0.51998 (14)	0.0244 (7)	
C29	0.0942 (5)	−0.1046 (4)	0.53744 (15)	0.0267 (8)	
C30	0.0450 (5)	0.1528 (4)	0.48204 (14)	0.0244 (7)	
C31	0.5852 (4)	0.0696 (4)	0.94649 (14)	0.0220 (7)	
C32	0.3907 (5)	0.0870 (4)	1.02952 (14)	0.0238 (7)	
C33	0.4753 (4)	0.1608 (4)	0.97543 (14)	0.0221 (7)	
C34	0.4474 (5)	0.3329 (4)	0.95307 (15)	0.0250 (7)	
H1	−0.06715	0.8156	0.85493	0.0299*	
H2	−0.0442	1.03605	0.77688	0.0333*	
H3	0.06352	1.00786	0.68481	0.0337*	
H25	0.32047	0.48804	0.97322	0.0385*	
H4	0.12896	0.76008	0.67241	0.0317*	
H5C	−0.15805	0.46729	0.92075	0.0329*	
H26	0.38949	0.05396	0.60649	0.0401*	
H6B	−0.05285	0.61312	0.92131	0.0329*	
H7A	0.02902	0.44235	0.9412	0.0329*	
H8	0.07957	0.01854	0.95149	0.0333*	
H9	0.12243	−0.23709	1.00075	0.0370*	
H10	0.41592	−0.06346	0.81974	0.0312*	
H11	0.45427	−0.31402	0.8739	0.0330*	
H12	0.59537	0.7028	0.60182	0.0363*	
H13	0.75282	0.80224	0.659	0.0473*	
H14	0.70869	0.70983	0.75891	0.0439*	
H15	0.49388	0.53435	0.80057	0.0350*	
H16B	0.38648	0.61772	0.55362	0.0349*	
H17A	0.2927	0.45945	0.56184	0.0349*	
H18C	0.49271	0.45663	0.56808	0.0349*	
H19	−0.07407	0.26017	0.58795	0.0319*	
H20	−0.29926	0.1537	0.561	0.0358*	
H21	−0.35117	0.22499	0.74466	0.0280*	
H22	−0.56868	0.11802	0.7132	0.0315*	
H23	0.15861	−0.17759	0.56312	0.0320*	
H24	0.31469	0.14613	1.05021	0.0285*	

### (1-Br) Bis{N-[1-(pyridin-2-yl-κN)ethylidene]pyridine-4-carbohydrazonato-κ2N',O}nickel(II)–2,5-dibromoterephthalic acid (1/1) Atomic displacement parameters (Å^2^)

**Table:**

	*U*^11^	*U*^22^	*U*^33^	*U*^12^	*U*^13^	*U*^23^
Br1	0.0421 (2)	0.02082 (19)	0.0378 (2)	−0.00217 (16)	0.00356 (18)	−0.00364 (16)
Br2	0.0364 (2)	0.02332 (19)	0.02352 (18)	0.00005 (15)	0.00553 (15)	−0.00413 (14)
Ni1	0.0290 (2)	0.0171 (2)	0.0152 (2)	0.00158 (18)	−0.00022 (17)	−0.00274 (16)
O1	0.0351 (14)	0.0193 (12)	0.0176 (11)	0.0045 (10)	0.0019 (10)	−0.0031 (9)
O2	0.0299 (13)	0.0218 (12)	0.0179 (11)	−0.0006 (10)	0.0003 (10)	−0.0045 (10)
O3	0.0476 (16)	0.0161 (12)	0.0278 (13)	0.0055 (12)	0.0054 (12)	−0.0038 (10)
O4	0.0522 (18)	0.0197 (13)	0.0363 (15)	0.0025 (12)	0.0146 (14)	−0.0004 (12)
O5	0.0342 (15)	0.0445 (16)	0.0210 (12)	−0.0107 (13)	−0.0038 (11)	−0.0044 (12)
O6	0.0393 (16)	0.0491 (18)	0.0258 (13)	−0.0160 (14)	−0.0021 (12)	−0.0004 (13)
N1	0.0258 (15)	0.0195 (14)	0.0189 (13)	0.0014 (12)	−0.0010 (12)	−0.0039 (11)
N2	0.0245 (15)	0.0157 (13)	0.0175 (13)	−0.0005 (11)	−0.0013 (11)	−0.0021 (11)
N3	0.0283 (15)	0.0148 (13)	0.0159 (13)	−0.0002 (12)	−0.0007 (11)	−0.0004 (11)
N4	0.0367 (18)	0.0179 (14)	0.0275 (15)	0.0020 (13)	−0.0031 (14)	−0.0036 (12)
N5	0.0259 (15)	0.0206 (14)	0.0217 (14)	0.0047 (12)	−0.0029 (12)	−0.0049 (12)
N6	0.0244 (15)	0.0191 (14)	0.0164 (13)	0.0043 (12)	−0.0006 (11)	−0.0026 (11)
N7	0.0266 (15)	0.0225 (14)	0.0173 (13)	0.0006 (12)	−0.0011 (12)	−0.0037 (11)
N8	0.0287 (16)	0.0276 (16)	0.0215 (14)	0.0007 (13)	−0.0018 (12)	−0.0057 (12)
C1	0.0215 (16)	0.0187 (16)	0.0200 (15)	−0.0002 (13)	−0.0012 (13)	−0.0052 (13)
C2	0.0281 (18)	0.0211 (17)	0.0241 (17)	0.0004 (14)	0.0027 (14)	−0.0063 (14)
C3	0.035 (2)	0.0180 (17)	0.0292 (18)	0.0049 (15)	−0.0018 (16)	−0.0063 (15)
C4	0.033 (2)	0.0217 (18)	0.0248 (18)	0.0036 (15)	−0.0010 (15)	0.0006 (14)
C5	0.0308 (19)	0.0233 (18)	0.0211 (17)	0.0041 (15)	0.0017 (15)	−0.0020 (14)
C6	0.0256 (17)	0.0180 (16)	0.0184 (15)	−0.0029 (14)	0.0019 (13)	−0.0043 (13)
C7	0.035 (2)	0.0220 (18)	0.0218 (17)	0.0014 (15)	0.0051 (15)	−0.0042 (14)
C8	0.0229 (17)	0.0190 (16)	0.0200 (16)	−0.0004 (14)	−0.0013 (13)	−0.0034 (13)
C9	0.0241 (17)	0.0182 (16)	0.0208 (16)	−0.0020 (14)	−0.0021 (13)	−0.0051 (13)
C10	0.035 (2)	0.0222 (18)	0.0251 (18)	0.0011 (15)	0.0012 (15)	−0.0066 (14)
C11	0.041 (2)	0.0214 (18)	0.0249 (18)	−0.0005 (16)	0.0032 (16)	0.0005 (15)
C12	0.034 (2)	0.0229 (18)	0.0210 (17)	−0.0011 (15)	−0.0023 (15)	−0.0060 (14)
C13	0.033 (2)	0.0233 (18)	0.0285 (18)	0.0050 (15)	−0.0060 (16)	−0.0106 (15)
C14	0.0260 (18)	0.0210 (17)	0.0207 (16)	0.0045 (14)	0.0005 (14)	−0.0042 (14)
C15	0.032 (2)	0.031 (2)	0.0248 (18)	−0.0023 (16)	0.0010 (16)	−0.0046 (16)
C16	0.031 (2)	0.045 (2)	0.042 (2)	−0.0127 (19)	0.0019 (18)	−0.010 (2)
C17	0.032 (2)	0.049 (3)	0.032 (2)	−0.0068 (19)	−0.0012 (17)	−0.0148 (19)
C18	0.031 (2)	0.033 (2)	0.0244 (18)	0.0048 (16)	−0.0049 (15)	−0.0091 (16)
C19	0.0244 (17)	0.0214 (17)	0.0184 (16)	0.0052 (14)	−0.0003 (13)	−0.0038 (13)
C20	0.033 (2)	0.032 (2)	0.0195 (17)	−0.0017 (16)	0.0017 (15)	−0.0043 (15)
C21	0.0292 (18)	0.0149 (15)	0.0209 (16)	0.0051 (14)	−0.0008 (14)	−0.0031 (13)
C22	0.0263 (18)	0.0161 (16)	0.0210 (16)	0.0041 (14)	−0.0010 (14)	−0.0045 (13)
C23	0.0277 (18)	0.0303 (19)	0.0202 (16)	0.0013 (15)	0.0037 (14)	−0.0076 (15)
C24	0.034 (2)	0.039 (2)	0.0184 (16)	0.0008 (17)	−0.0017 (15)	−0.0106 (16)
C25	0.0300 (18)	0.0196 (17)	0.0186 (16)	0.0046 (14)	−0.0011 (14)	−0.0042 (13)
C26	0.0282 (19)	0.0246 (18)	0.0225 (17)	0.0030 (15)	−0.0000 (14)	−0.0024 (14)
C27	0.033 (2)	0.0277 (19)	0.0251 (18)	−0.0006 (16)	−0.0020 (16)	−0.0074 (15)
C28	0.0291 (18)	0.0257 (18)	0.0173 (16)	−0.0005 (15)	0.0004 (14)	−0.0055 (14)
C29	0.033 (2)	0.0238 (18)	0.0199 (16)	0.0031 (15)	−0.0004 (15)	−0.0018 (14)
C30	0.0312 (19)	0.0193 (16)	0.0196 (16)	−0.0017 (14)	0.0052 (14)	−0.0031 (13)
C31	0.0258 (17)	0.0213 (17)	0.0191 (15)	0.0005 (14)	−0.0003 (13)	−0.0071 (13)
C32	0.0287 (18)	0.0232 (17)	0.0201 (16)	−0.0003 (14)	−0.0010 (14)	−0.0077 (14)
C33	0.0276 (18)	0.0188 (16)	0.0201 (16)	−0.0018 (14)	−0.0027 (14)	−0.0049 (13)
C34	0.0311 (19)	0.0182 (17)	0.0254 (17)	0.0026 (15)	−0.0055 (15)	−0.0050 (14)

### (1-Br) Bis{N-[1-(pyridin-2-yl-κN)ethylidene]pyridine-4-carbohydrazonato-κ2N',O}nickel(II)–2,5-dibromoterephthalic acid (1/1) Geometric parameters (Å, º)

**Table:**

Br1—C30	1.890 (3)	C16—C17	1.373 (6)
Br2—C31	1.906 (3)	C17—C18	1.392 (5)
Ni1—O1	2.079 (2)	C19—C20	1.488 (5)
Ni1—O2	2.118 (3)	C21—C22	1.506 (5)
Ni1—N1	2.110 (3)	C22—C23	1.395 (5)
Ni1—N2	1.986 (3)	C22—C25	1.394 (5)
Ni1—N5	2.116 (3)	C23—C24	1.378 (6)
Ni1—N6	1.983 (3)	C25—C26	1.385 (6)
O1—C8	1.272 (4)	C27—C28	1.508 (6)
O2—C21	1.279 (4)	C28—C29	1.399 (5)
C34—O3	1.324 (4)	C28—C30	1.391 (5)
C34—O4	1.207 (4)	C29—C30^i^	1.388 (6)
C27—O5	1.300 (4)	C31—C32^ii^	1.385 (5)
C27—O6	1.223 (4)	C31—C33	1.394 (5)
N1—C1	1.354 (5)	C32—C33	1.406 (4)
N1—C5	1.339 (4)	C33—C34	1.509 (5)
N2—N3	1.380 (3)	O3—H25	0.840
N2—C6	1.290 (4)	O5—H26	0.840
N3—C8	1.337 (5)	C2—H1	0.950
N4—C11	1.339 (5)	C3—H2	0.950
N4—C13	1.338 (4)	C4—H3	0.950
N5—C14	1.364 (4)	C5—H4	0.950
N5—C18	1.340 (5)	C7—H5C	0.980
N6—N7	1.380 (4)	C7—H6B	0.980
N6—C19	1.297 (4)	C7—H7A	0.980
N7—C21	1.332 (4)	C10—H8	0.950
N8—C24	1.343 (4)	C11—H9	0.950
N8—C26	1.344 (5)	C12—H10	0.950
C1—C2	1.391 (5)	C13—H11	0.950
C1—C6	1.484 (4)	C15—H12	0.950
C2—C3	1.394 (4)	C16—H13	0.950
C3—C4	1.379 (5)	C17—H14	0.950
C4—C5	1.392 (5)	C18—H15	0.950
C6—C7	1.485 (5)	C20—H16B	0.980
C8—C9	1.499 (4)	C20—H17A	0.980
C9—C10	1.391 (4)	C20—H18C	0.980
C9—C12	1.383 (5)	C23—H19	0.950
C10—C11	1.387 (5)	C24—H20	0.950
C12—C13	1.384 (5)	C25—H21	0.950
C14—C15	1.394 (5)	C26—H22	0.950
C14—C19	1.482 (6)	C29—H23	0.950
C15—C16	1.387 (7)	C32—H24	0.950
			
O1—Ni1—O2	90.25 (9)	N8—C26—C25	122.2 (3)
O1—Ni1—N1	154.75 (11)	O5—C27—O6	126.1 (4)
O1—Ni1—N2	77.64 (10)	O5—C27—C28	111.6 (3)
O1—Ni1—N5	96.88 (10)	O6—C27—C28	122.3 (3)
O1—Ni1—N6	103.71 (11)	C27—C28—C29	118.1 (3)
O2—Ni1—N1	97.70 (11)	C27—C28—C30	124.1 (3)
O2—Ni1—N2	97.26 (10)	C29—C28—C30	117.7 (4)
O2—Ni1—N5	155.41 (10)	C28—C29—C30^i^	121.3 (3)
O2—Ni1—N6	77.36 (10)	Br1—C30—C28	122.5 (3)
N1—Ni1—N2	77.60 (11)	Br1—C30—C29^i^	116.2 (2)
N1—Ni1—N5	85.78 (11)	C28—C30—C29^i^	121.1 (3)
N1—Ni1—N6	101.41 (11)	Br2—C31—C32^ii^	115.4 (3)
N2—Ni1—N5	107.23 (12)	Br2—C31—C33	123.7 (2)
N2—Ni1—N6	174.40 (12)	C32^ii^—C31—C33	121.0 (3)
N5—Ni1—N6	78.10 (12)	C31^ii^—C32—C33	121.9 (3)
Ni1—O1—C8	108.6 (2)	C31—C33—C32	117.1 (3)
Ni1—O2—C21	107.8 (2)	C31—C33—C34	124.0 (3)
Ni1—N1—C1	112.86 (19)	C32—C33—C34	118.8 (3)
Ni1—N1—C5	127.7 (2)	O3—C34—O4	123.7 (3)
C1—N1—C5	118.7 (3)	O3—C34—C33	112.6 (3)
Ni1—N2—N3	117.9 (2)	O4—C34—C33	123.7 (3)
Ni1—N2—C6	120.5 (2)	C34—O3—H25	109.465
N3—N2—C6	120.7 (3)	C27—O5—H26	109.470
N2—N3—C8	107.6 (2)	C1—C2—H1	120.447
C11—N4—C13	117.1 (3)	C3—C2—H1	120.444
Ni1—N5—C14	110.8 (2)	C2—C3—H2	120.318
Ni1—N5—C18	127.9 (2)	C4—C3—H2	120.314
C14—N5—C18	118.8 (3)	C3—C4—H3	120.872
Ni1—N6—N7	118.25 (18)	C5—C4—H3	120.856
Ni1—N6—C19	120.4 (3)	N1—C5—H4	118.484
N7—N6—C19	121.3 (3)	C4—C5—H4	118.485
N6—N7—C21	108.4 (3)	C6—C7—H5C	109.473
C24—N8—C26	118.2 (3)	C6—C7—H6B	109.467
N1—C1—C2	121.4 (3)	C6—C7—H7A	109.477
N1—C1—C6	114.9 (3)	H5C—C7—H6B	109.476
C2—C1—C6	123.6 (3)	H5C—C7—H7A	109.472
C1—C2—C3	119.1 (3)	H6B—C7—H7A	109.463
C2—C3—C4	119.4 (3)	C9—C10—H8	120.656
C3—C4—C5	118.3 (3)	C11—C10—H8	120.656
N1—C5—C4	123.0 (3)	N4—C11—H9	118.224
N2—C6—C1	113.0 (3)	C10—C11—H9	118.224
N2—C6—C7	124.4 (3)	C9—C12—H10	120.307
C1—C6—C7	122.5 (3)	C13—C12—H10	120.297
O1—C8—N3	127.7 (3)	N4—C13—H11	118.377
O1—C8—C9	117.4 (3)	C12—C13—H11	118.380
N3—C8—C9	114.9 (3)	C14—C15—H12	120.593
C8—C9—C10	121.0 (3)	C16—C15—H12	120.579
C8—C9—C12	120.9 (3)	C15—C16—H13	120.167
C10—C9—C12	118.0 (3)	C17—C16—H13	120.138
C9—C10—C11	118.7 (3)	C16—C17—H14	120.526
N4—C11—C10	123.6 (3)	C18—C17—H14	120.526
C9—C12—C13	119.4 (3)	N5—C18—H15	118.856
N4—C13—C12	123.2 (3)	C17—C18—H15	118.858
N5—C14—C15	121.4 (4)	C19—C20—H16B	109.483
N5—C14—C19	115.4 (3)	C19—C20—H17A	109.479
C15—C14—C19	123.3 (3)	C19—C20—H18C	109.470
C14—C15—C16	118.8 (3)	H16B—C20—H17A	109.459
C15—C16—C17	119.7 (4)	H16B—C20—H18C	109.470
C16—C17—C18	118.9 (4)	H17A—C20—H18C	109.466
N5—C18—C17	122.3 (3)	C22—C23—H19	120.672
N6—C19—C14	112.8 (3)	C24—C23—H19	120.657
N6—C19—C20	125.3 (4)	N8—C24—H20	118.393
C14—C19—C20	121.9 (3)	C23—C24—H20	118.385
O2—C21—N7	127.6 (3)	C22—C25—H21	120.311
O2—C21—C22	118.3 (3)	C26—C25—H21	120.324
N7—C21—C22	114.1 (3)	N8—C26—H22	118.897
C21—C22—C23	120.6 (3)	C25—C26—H22	118.896
C21—C22—C25	121.1 (3)	C28—C29—H23	119.375
C23—C22—C25	118.3 (3)	C30^i^—C29—H23	119.374
C22—C23—C24	118.7 (3)	C31^ii^—C32—H24	119.053
N8—C24—C23	123.2 (4)	C33—C32—H24	119.046
C22—C25—C26	119.4 (3)		
			
O1—Ni1—O2—C21	−98.39 (14)	Ni1—N6—N7—C21	7.7 (3)
O2—Ni1—O1—C8	−91.08 (16)	Ni1—N6—C19—C14	−1.8 (3)
O1—Ni1—N1—C1	−13.5 (4)	Ni1—N6—C19—C20	177.56 (19)
O1—Ni1—N1—C5	156.50 (19)	N7—N6—C19—C14	178.4 (2)
N1—Ni1—O1—C8	17.8 (3)	N7—N6—C19—C20	−2.2 (5)
O1—Ni1—N2—N3	−7.43 (18)	C19—N6—N7—C21	−172.6 (3)
O1—Ni1—N2—C6	−176.7 (2)	N6—N7—C21—O2	−2.2 (4)
N2—Ni1—O1—C8	6.30 (16)	N6—N7—C21—C22	178.0 (2)
O1—Ni1—N5—C14	115.31 (17)	C24—N8—C26—C25	0.2 (5)
O1—Ni1—N5—C18	−83.1 (2)	C26—N8—C24—C23	−0.0 (5)
N5—Ni1—O1—C8	112.50 (17)	N1—C1—C2—C3	−3.3 (5)
O1—Ni1—N6—N7	79.58 (19)	N1—C1—C6—N2	9.9 (4)
O1—Ni1—N6—C19	−100.16 (19)	N1—C1—C6—C7	−170.5 (3)
N6—Ni1—O1—C8	−168.13 (16)	C2—C1—C6—N2	−167.5 (3)
O2—Ni1—N1—C1	93.81 (18)	C2—C1—C6—C7	12.1 (5)
O2—Ni1—N1—C5	−96.2 (2)	C6—C1—C2—C3	173.9 (3)
N1—Ni1—O2—C21	105.65 (14)	C1—C2—C3—C4	0.6 (5)
O2—Ni1—N2—N3	81.23 (19)	C2—C3—C4—C5	2.1 (5)
O2—Ni1—N2—C6	−88.0 (2)	C3—C4—C5—N1	−2.5 (6)
N2—Ni1—O2—C21	−175.96 (14)	O1—C8—C9—C10	−161.1 (3)
O2—Ni1—N5—C14	9.4 (4)	O1—C8—C9—C12	16.4 (5)
O2—Ni1—N5—C18	171.00 (16)	N3—C8—C9—C10	17.2 (5)
N5—Ni1—O2—C21	8.9 (3)	N3—C8—C9—C12	−165.3 (3)
O2—Ni1—N6—N7	−7.56 (16)	C8—C9—C10—C11	177.1 (3)
O2—Ni1—N6—C19	172.7 (2)	C8—C9—C12—C13	−178.0 (3)
N6—Ni1—O2—C21	5.61 (14)	C10—C9—C12—C13	−0.4 (5)
N1—Ni1—N2—N3	177.6 (2)	C12—C9—C10—C11	−0.5 (5)
N1—Ni1—N2—C6	8.3 (2)	C9—C10—C11—N4	0.5 (6)
N2—Ni1—N1—C1	−1.98 (18)	C9—C12—C13—N4	1.5 (6)
N2—Ni1—N1—C5	168.0 (3)	N5—C14—C15—C16	0.4 (5)
N1—Ni1—N5—C14	−89.92 (18)	N5—C14—C19—N6	13.5 (4)
N1—Ni1—N5—C18	71.7 (2)	N5—C14—C19—C20	−165.9 (3)
N5—Ni1—N1—C1	−110.67 (19)	C15—C14—C19—N6	−165.1 (3)
N5—Ni1—N1—C5	59.3 (2)	C15—C14—C19—C20	15.5 (5)
N1—Ni1—N6—N7	−103.00 (18)	C19—C14—C15—C16	178.9 (3)
N1—Ni1—N6—C19	77.3 (2)	C14—C15—C16—C17	2.2 (6)
N6—Ni1—N1—C1	172.39 (18)	C15—C16—C17—C18	−2.6 (6)
N6—Ni1—N1—C5	−17.6 (3)	C16—C17—C18—N5	0.5 (6)
N2—Ni1—N5—C14	−165.54 (16)	O2—C21—C22—C23	−176.1 (2)
N2—Ni1—N5—C18	−3.9 (2)	O2—C21—C22—C25	3.5 (4)
N5—Ni1—N2—N3	−100.89 (19)	N7—C21—C22—C23	3.7 (4)
N5—Ni1—N2—C6	89.8 (2)	N7—C21—C22—C25	−176.7 (2)
N5—Ni1—N6—N7	173.9 (2)	C21—C22—C23—C24	179.7 (3)
N5—Ni1—N6—C19	−5.89 (18)	C21—C22—C25—C26	−179.6 (2)
N6—Ni1—N5—C14	12.69 (16)	C23—C22—C25—C26	0.0 (4)
N6—Ni1—N5—C18	174.3 (2)	C25—C22—C23—C24	0.1 (4)
Ni1—O1—C8—N3	−5.3 (4)	C22—C23—C24—N8	−0.1 (5)
Ni1—O1—C8—C9	172.72 (19)	C22—C25—C26—N8	−0.2 (5)
Ni1—O2—C21—N7	−3.6 (4)	O5—C27—C28—C29	−43.0 (4)
Ni1—O2—C21—C22	176.19 (17)	O5—C27—C28—C30	139.8 (3)
Ni1—N1—C1—C2	174.0 (2)	O6—C27—C28—C29	136.1 (3)
Ni1—N1—C1—C6	−3.4 (3)	O6—C27—C28—C30	−41.1 (5)
Ni1—N1—C5—C4	−169.6 (2)	C27—C28—C29—C30^i^	−177.8 (3)
C1—N1—C5—C4	−0.1 (5)	C27—C28—C30—Br1	−8.3 (5)
C5—N1—C1—C2	3.0 (5)	C27—C28—C30—C29^i^	177.6 (3)
C5—N1—C1—C6	−174.4 (3)	C29—C28—C30—Br1	174.6 (3)
Ni1—N2—N3—C8	6.7 (3)	C29—C28—C30—C29^i^	0.4 (5)
Ni1—N2—C6—C1	−12.3 (4)	C30—C28—C29—C30^i^	−0.4 (5)
Ni1—N2—C6—C7	168.1 (2)	C28—C29—C30^i^—Br1^i^	174.9 (3)
N3—N2—C6—C1	178.7 (3)	C28—C29—C30^i^—C28^i^	0.5 (5)
N3—N2—C6—C7	−0.9 (5)	Br2—C31—C32^ii^—C33^ii^	179.7 (2)
C6—N2—N3—C8	176.0 (3)	Br2—C31—C33—C32	−179.7 (2)
N2—N3—C8—O1	−0.5 (5)	Br2—C31—C33—C34	−1.8 (5)
N2—N3—C8—C9	−178.5 (2)	C32^ii^—C31—C33—C32	−0.2 (5)
C11—N4—C13—C12	−1.5 (6)	C32^ii^—C31—C33—C34	177.7 (3)
C13—N4—C11—C10	0.5 (6)	C33—C31—C32^ii^—C33^ii^	0.2 (6)
Ni1—N5—C14—C15	161.1 (2)	C31^ii^—C32—C33—C31	0.2 (5)
Ni1—N5—C14—C19	−17.6 (3)	C31^ii^—C32—C33—C34	−177.8 (3)
Ni1—N5—C18—C17	−158.3 (2)	C31—C33—C34—O3	177.0 (3)
C14—N5—C18—C17	2.0 (5)	C31—C33—C34—O4	−4.1 (6)
C18—N5—C14—C15	−2.4 (5)	C32—C33—C34—O3	−5.2 (5)
C18—N5—C14—C19	178.9 (3)	C32—C33—C34—O4	173.8 (3)

Symmetry codes: (i) −*x*, −*y*, −*z*+1; (ii) −*x*+1, −*y*, −*z*+2.

### (1-Br) Bis{N-[1-(pyridin-2-yl-κN)ethylidene]pyridine-4-carbohydrazonato-κ2N',O}nickel(II)–2,5-dibromoterephthalic acid (1/1) Hydrogen-bond geometry (Å, º)

**Table:**

*D*—H···*A*	*D*—H	H···*A*	*D*···*A*	*D*—H···*A*
O3—H25···N4^iii^	0.84	1.87	2.706 (4)	178
O5—H26···N8^iv^	0.84	1.72	2.557 (4)	172

Symmetry codes: (iii) *x*, *y*+1, *z*; (iv) *x*+1, *y*, *z*.
